# Midwives’ perspectives of their ability to promote the oral health of pregnant women in Victoria, Australia

**DOI:** 10.1186/s12884-015-0536-x

**Published:** 2015-05-07

**Authors:** Adina Y Heilbrunn-Lang, Andrea M de Silva, Gillian Lang, Ajesh George, Allison Ridge, Maree Johnson, Sameer Bhole, Carole Gilmour

**Affiliations:** Centre for Applied Oral Health Research, Dental Health Services Victoria, Melbourne, Australia; Health Promotion, Dental Health Services Victoria, Melbourne, Australia; Centre for Applied Nursing Research, University of Western Sydney/ South Western Sydney Local Health District, Sydney, Australia; Ingham Institute Applied Medical Research, Sydney, Australia; Faculty of Dentistry, University of Sydney, Sydney, Australia; Faculty of Health Sciences, Australian Catholic University, Sydney, Australia; Sydney Local Health District Oral Health Services and Sydney Dental Hospital, Sydney, Australia; Australian College of Midwifery Victorian Branch, Melbourne, Australia; Monash University, Melbourne, Australia

**Keywords:** Oral health, Pregnancy, Midwives, Antenatal care, Education program

## Abstract

**Background:**

Midwives have a potential role in promoting the oral health of pregnant women although they have little formal training in this area. The aim of this study was to explore the perspectives of midwives in Victoria towards incorporating oral health promotion into their antenatal practice after undergoing training through the Midwifery Initiated Oral Health (MIOH) online education program.

**Methods:**

A purposive sample of thirty-nine midwives from maternity services across Victoria, Australia were invited to participate in an online MIOH education program in October 2012. The program included three self-paced modules covering oral health screening, referral processes, and theoretical and practical skill assessments. A mixed methods design was used to capture midwives perspectives. Evaluation questionnaires, completed pre- and post-training, captured knowledge and confidence (confidence likert scale), and also included five opened-ended questions post-training. Open-ended questions, feedback forms and unsolicited emails formed the data for qualitative analysis. Data were analysed using content and thematic analysis and descriptive statistics.

**Results:**

Thirty-three midwives completed the MIOH education program and demonstrated a significant increase (51.5%) in their confidence to promote oral health. All participants viewed the program as suitable, acceptable and useful for their practice and were happy to recommend the course to other Victorian midwives. Participants indicated that it would be feasible to incorporate oral health into the first antenatal booking visit and recognised that oral health promotion was within their scope of practice.

**Conclusions:**

This study has shown that the MIOH education program is a valued resource that can assist midwives to increase their confidence and skills to incorporate oral health promotion into their practice. A key barrier identified was time constraints during antenatal care booking visits. However, it is evident that with relevant training it would be feasible and acceptable for Victorian midwives to incorporate oral health promotion within their practice. The current engagement with midwives in Victoria and other parts of Australia provides an opportunity to continue to explore and define the role of antenatal health care professionals in oral health promotion at a state and national level.

## Background

A growing body of evidence suggests that poor oral health of women during pregnancy negatively impacts health outcomes for both the mother and baby [1-3]. Early childhood caries is a significant issue in Victoria, Australia, with almost half of all six-year-old children having a history of tooth decay [4]. Poor oral health can negatively affect a child’s speech, growth, learning, development, self-esteem, social and psychological wellbeing and influence the development of lifetime habits [4-9]. Fortunately, oral disease is mostly preventable with appropriate oral hygiene knowledge, skills and practices [10,11]. Pregnancy is an important time for women to care for their oral health because of the increased susceptibility to periodontal (gum) infection and the links between advanced gum disease and premature birth and low birth weight babies [12-15]. In addition poor maternal oral health may lead to adverse impacts upon the unborn child’s oral health outcomes such as the transfer of maternal cariogenic bacterial flora to the child [16,17].

In Victoria, approximately 70 percent of hospital-based births occur through public hospitals, the majority in Metropolitan Melbourne, and the remainder are in private hospitals [18]. Through public services, women experiencing a normal pregnancy are usually managed by local midwives and general practitioners and can choose from several models of care [18]. Public hospital services are subsidised by the Australian government Medicare system (private practitioners often charge extra).

Victorian state government policy provides priority access (offering the next available appointment) to public dental services for pregnant women with a health care card for a small co-payment (reduced fee) for public dental care since 2007 [19]. Health care cards are provided to eligible Australians (for example low-income earners, people experiencing sickness or disability, carers, students, elderly) though the Federal Government welfare system [20]. However, uptake of dental services and treatment among pregnant women remains relatively low, particularly among those at high risk of poor oral health (e.g. health care card holders) [21]. It is reported that women often do not seek dental advice or treatment during pregnancy and underlying deterrents include barriers to access, capacity to pay, education, socio-economic status, lack of community awareness about the importance of oral health during pregnancy and misconceptions as to the safety of dental treatment in pregnancy [21-23]. In addition, historically, the lack of international consensus evidence and clinical guidelines regarding the management of oral health during pregnancy renders some clinicians hesitant to treat pregnant women [1,24]. However in Victoria, the public dental service, Dental Health Services Victoria (DHSV), has developed clinical guidelines for dental management of pregnant patients to address this [25].

Prevention and early detection of oral disease in pregnancy can assist in managing a significant bacterial and inflammatory condition during pregnancy (periodontitis) and reduce the risk of infants developing early childhood caries [26]. Educating pregnant women on the importance of dental care and good oral hygiene practices and the safety of receiving dental treatment during pregnancy is essential [22,27]. Therefore, internationally research and oral health care and maternity care guidelines have begun to recognise the role of antenatal care providers in promoting the oral health of pregnant women [26,28,29]. More recently the revised Australian antenatal care guidelines (Clinical Practice Guidelines: Antenatal care Module 1) [30] have incorporated oral health promotion as part of the first antenatal booking visit.

A number of countries such as the United States of America, Iran and Australia have identified the role of midwives in oral health care [1,21,22,31-36]. Prior research has established that midwives conducting the first antenatal booking visit are well placed to provide oral health assessment, education and referral [26,28,37,38]. Preliminary Australian research conducted with midwives in Southwest Sydney demonstrated their recognition of the role that they can play in promoting the oral health of pregnant women. The study highlighted current barriers including gaps in the oral health knowledge, skills and confidence of midwives; the lack of available oral health education for midwives; the frequency of oral disease among pregnant women observed by the midwives; the lack of structure for incorporating oral health assessment and referral pathways; and limited time available to incorporate oral health assessment into the booking visit [1,23,39]. Sensitivity toward the oral health of pregnant women was also mentioned and access barriers to dental treatment including the cost of treatment and confusion about safety of dental treatment among dental professionals.

Australian and other international intervention research on oral health and pregnancy appears to be predominantly focused on educating pregnant women using various resources such as DVDs, PowerPoint presentations and printed materials rather than education of midwives in oral health promotion, assessment and referral [1,10,21,22,31-35,40,41].

In response to these identified gaps and a request for educational support by midwives, the Midwifery Initiated Oral Health (MIOH) online education program was developed and piloted in 2010 with midwives in New South Wales (NSW), Australia. The program included three self-paced modules covering oral health screening, referral processes, and theoretical and practical skill assessments [37,42]. Consultations and enquiry into oral health promotion among Victorian midwives revealed a similar educational gap, and led to the current study piloting the use of the MIOH online education program in the Victorian context and further investigation into the role of Victorian Midwives in oral health promotion.

The aim of this study was to explore the perspectives of midwives in Victoria towards incorporating oral health promotion into their antenatal practice after undergoing training through the Midwifery Initiated Oral Health (MIOH) online education program.

A number of key questions are addressed including:

1. What are Victorian midwives perspectives of the usefulness of the MIOH education program for informing antenatal practice?
2. What are midwives perspectives on the feasibility of incorporating oral health promotion into their antenatal practice after undergoing training?
3. Are midwives more confident in promoting oral health in their antenatal practice after undergoing training?

## Methods

### Study design

A mixed methods design with a predominantly descriptive qualitative element was used to capture midwives perspectives. Open-ended questions, feedback forms and unsolicited emails formed the data for qualitative analysis while a short confidence likert scale provided quantitative data for analysis.

### Sampling and participants

Victorian hospitals and maternity services were selected purposively to ensure that a range of hospitals and midwives were represented in the pilot study. Included hospitals and services were those that were identified as having high numbers of births or high antenatal booking visits in the first trimester (to ensure large numbers of women were being seen in the first antenatal visit). A selection of a range of metropolitan, rural and regional hospitals were approached as well as those identified in geographical areas recognised as having higher risk of poor oral health. Community health services including Koori Maternity Services (KMS) (a culturally appropriate maternity service for indigenous Australians – Aboriginal/ Koori and Torres Strait Islander women) midwives were also included as a priority group as they service a population at high risk of oral disease. The KMS midwives were invited through the Victorian Aboriginal Community Controlled Health Organisation, the peak body for Aboriginal Community Controlled Health Organisations in Victoria, Australia. Hospitals or services that did not meet these criteria were excluded. Midwives who did not conduct the first antenatal booking visit were also excluded.

### Recruitment

Five hospitals and the Victorian Aboriginal Community Controlled Health Services (representing thirteen KMS) were contacted via telephone, email and face-to-face meetings and invited to participate in the pilot study. An explanation of the nature and importance of the MIOH education program was provided for the management staff of identified hospitals and services by the program coordinator. Management staff subsequently informed appropriate midwives (involved in conducting the first antenatal booking visit) of the program. Participation was open to all midwives involved with the first antenatal booking visit. Midwives were invited and elected to participate through an expression of interest form. Recruitment was undertaken until sufficient representation of a range of midwifery hospitals and services was obtained for the pilot and expression of interest received prior to the cut-off date. In addition, four midwives involved in conducting the first antenatal booking visit expressed interest and participated (these midwives represented four maternity services that had not been contacted to participate).

### The intervention: MIOH education program

The MIOH education program was initially developed and piloted with midwives practising in NSW. The program was developed under the auspices of the Centre for Applied Nursing Research, Sydney and South Western Local Health Districts and the University of Western Sydney in consultation with a panel of professional experts including midwives, nurses, dentists and academics to provide midwives with knowledge and skills in oral health promotion [37]. In partnership with DHSV the education program was adapted and piloted with Victorian midwives as part of the Healthy Families, Healthy Smiles (HFHS) initiative funded by the Victorian Government. The evidence-based MIOH education program aims to increase the oral health related knowledge and confidence of midwives involved in antenatal care and consists of three self-paced modules focusing on oral health screening and referral and a competency assessment of theoretical knowledge and practical skill. Participants were given a three month period in which to complete the program. The course was designed to be completed over 16 hours and was endorsed by the Australian College of Midwives as a continuing professional development (CPD) activity awarding midwives 16 CPD points on successful completion (full detail of the MIOH education program and development reported elsewhere [37]).

### Data collection

All midwives were invited to complete an online pre-questionnaire upon being enrolled as a participant in the study. Questionnaires were developed based on the literature and were trialled with a small sample of midwives in NSW and further tested as part of our pilot study [42]. Participants were provided with access to the MIOH education program on completion of the pre-questionnaire.

The post-questionnaire was administered to participants who successfully completed the online education program. Both pre/post-questionnaires examined knowledge (24 items) and confidence (7 items involving a 3 point likert scales – confident, somewhat confident and not confident) before and after completing the MIOH education program using the same questions. The post-questionnaire included the following five additional open-ended items: What did you find most useful about this education program?; Would you recommend this education program to other Victorian Midwives?; Do you think it would it be feasible to include oral health in the first antenatal booking visit?; Do you have any suggestions to improve this education program?; Do you have any other comments?

Data from pre/post-questionnaires were supplemented by feedback forms and supportive emails. The feedback form asked participants their perceptions of the best aspects of the program, what could be improved, and any other comments they wished to make. Unsolicited emails received by the program coordinator from participating midwives were collected and provided to the research team for reference.

This paper reports on changes in self-reported confidence (pre/post-questionnaire) and the qualitative component of the pilot study (open-ended post-questionnaire responses, feedback forms and additional ad hoc feedback received). Changes in oral health knowledge and post confidence levels were collated with NSW data and will be reported elsewhere (George A, Lang G, Johnson M, Ridge A, de Silva A, Ajwani S, Bhole S, Blinkhorn A, Dahlen H, Ellis S, Yeo A, Langdon R, Carpenter L, Heilbrunn A: Evaluation of an Oral Health Education Program for Midwives, submitted).

### Data analysis

Data from the five post-questionnaire open-ended items were entered into Nvivo10 [43] and analysed. To develop a broad understanding of the range of participant perspectives an initial content analysis was conducted for each questionnaire item. Responses were then coded and categorised within the pre-determined items, followed by thematic analysis, re-analysis of the overarching and overlapping common emerging themes [44]. Emerging themes initially derived from questionnaire responses were re-analysed accompanied by a comprehensive analysis of all data including feedback forms and supporting emails. Data were then re-coded, categorised and analysed by a second researcher and reviewed by the research team to reach consensus, adding rigour to the analysis through peer coding [45]. Pseudonyms were allocated to participants to maintain confidentiality and anonymity.

Data from the pre/post-questionnaire confidence likert scales were analysed using IBM SPSS Statistics for Windows, Version 21.0 (IBM Corp, Armonk, NY, USA). T-tests were used to determine the differences between the two test times and assess whether there were any significant changes in the dependent variable (confidence level) due to the independent variable (completion of education program).

### Ethical considerations

Ethics approval was obtained from Dental Health Services Victoria Human Research Ethics Committee (HREC #236). Participation was voluntary and privacy and confidentiality of all study information was maintained. Only anonymous data was received and no individual results were reported. Consent for participation and publication of the research findings was implied on return of the completed online questionnaires.

## Results

Victorian midwives (n = 39) were purposively recruited into the study in October 2012. Thirty-three midwives (85% response rate) completed the MIOH education program and pre/post-questionnaires (n = 3 withdrew during the course and n = 3 did not complete the program in the study time frame). Additional feedback forms were received from six midwives and emails with additional feedback were received from eight midwives.

### Participant demographics

Participants worked in diverse settings including metropolitan and rural hospitals, and community health services (including Koori Maternity Services). The average age of participating midwives was 44.6 (±10.7) years old with 15.8 (±9.4) years of experience. Sixty-seven per cent (n = 22) of midwives were located in inner and outer metropolitan maternity services and 33% (n = 11) were from rural and regional maternity services.

Participants in the current study reflect similar characteristics to the wider midwifery population in Victoria, representing an ageing population, with the majority being females over the age of 45 years old. Overall the current cohort represented a similar range of number of years practising with the majority practising for ±15 years [46].

### Qualitative findings

Open-ended responses to qualitative questionnaire items, feedback forms and supporting emails provided rich data on the perspective of Victorian midwives on the usefulness of the MIOH education program and the feasibility of incorporating oral health promotion into antenatal care.

An analysis of the data revealed a number of emerging themes which have been discussed against the main study aim and research questions (see Table 1).

**Table 1:** **Study aims and key themes**

**Study aims**	**Themes**
Usefulness of an online oral health education program	• *Knowledge gap*
	• *Knowledge gains - oral health in pregnancy*
	• *Relevant content*
Feasibility of incorporating oral health promotion into antenatal practice	• *The role of the midwife in promoting oral health*
	• *Relevance to midwifery practice*
	• *Feasibility of incorporating oral health into antenatal care*
	• *Intentions to change practice*

#### Usefulness of the MIOH education program

##### Knowledge gap

On completion of the MIOH course midwives identified and discussed a gap in their prior knowledge and understanding of the potential impact of oral health on pregnancy outcomes for the mother and baby, and the lack of awareness of the importance of maternal oral health.

An absence of formal education was raised by a few midwives noting that oral health education was not taught in their midwifery training courses and highlighting the need for this to be addressed.

*“It [the MIOH education program] was incredibly valuable and I am quite astonished at my lack of knowledge prior to the program. I will certainly be ensuring that the midwives who conduct antenatal care are given the evidence and information that they should be giving to women.” (M10)*

*“I thought [the MIOH education program] was great. The learning program was relevant to both my practice as a midwife and maternal and child health nurse. Perhaps naively, I had no idea about vertical transmission of the bacteria contributing to early childhood caries.” (M Email 8)*

*“I have had discussions with my colleague at [the] University [about] how we can incorporate the MIOH Education program into [our] curriculum.” (M Email 5)*

##### Knowledge gains - oral health in pregnancy

One of the most useful aspects of the education program described by the midwives was the oral health knowledge and understanding it provided. In particular midwives referred to new knowledge gained on the physiological changes during pregnancy and the influence on oral health and pregnancy outcomes, and the links between mother and infant oral health, such as the transmission of streptococci from mother to child.

*“I found it [the MIOH education program] all useful but particularly the knowledge regarding pregnancy specific oral care and the transmission of mutans streptococci to infants from the mother. I really had no idea it wasn’t safe to brush teeth after vomiting.” (M10)*

*“The program… was very interesting and has increased my knowledge of oral care during pregnancy and particularly prevention of dental caries in pregnancy and beyond i.e. ECC [early childhood caries]. It has demonstrated the importance of educating women in this area and increasing public awareness, disputing myths and poor dental practices.” (M Feedback 4)*

##### Relevant content

Many of the midwives referred to the relevance and usefulness of the program content and resources provided e.g. evidence based journal articles.

*“This course has improved my knowledge about oral health in pregnancy, vastly. It was comprehensive, easily understood and hugely important to pregnant women and their families.” (M22)*

*“As a childbirth educator, I found this program extremely relevant. The reading material was informative and interesting.” (M9)*

*“The knowledge is vital in being able to perform a comprehensive antenatal assessment as well as being able to give advice that potentially improves dental health in the mother and her offspring.” (M10)*

*“The learning package was clear, self-explanatory and evidence [based].” (M34)*

#### Feasibility of incorporating oral health promotion into antenatal practice

##### The roles of the midwife in promoting oral health

Midwives highlighted that the education program provided a clarified and fresh understanding of the role of the midwife in oral health promotion. Midwives described their role as “*pivotal*” and providing a “*prime opportunity*” to promote oral health. This enthusiasm toward their new knowledge and practical skill was further reflected by a few participants who referred to taking steps to incorporate oral health into their practice.

*“I work in a community-based midwifery clinic in a low socioeconomic area; there is so much we can do as midwives to assist women. If it wasn’t for a pregnancy, these women would not normally access health care services and yet many of them do not have the knowledge to maintain their health, including dental care.” (M Email 6)*

*“Acknowledging the important role and the prime opportunities midwives have in educating women in optimising maternal and foetal wellbeing [was a most useful aspect of the education program].” (M1)*

##### Relevance to midwifery practice

All midwives indicated that they would recommend the MIOH education program to other Victorian midwives. Respondents used strong positive language and frequently acknowledged the importance of good oral health and its relevance for midwifery practice. Midwives described the oral health information and knowledge as “*valuable*”, “*vital*”, “*comprehensive*” and “*extremely important*” providing “*immense*” gains for pregnant women. Midwives mentioned that their main concern was for the wellbeing of their patient and recognised oral health promotion as being of obvious relevance to their role.

*“It’s amazing that oral health hasn’t been brought into antenatal care before now in this more structured fashion. I feel very passionate about it now. I have seen women with dental problems before particularly those with drug/ alcohol dependencies and I haven’t known how to approach the topic as I didn’t want to ‘embarrass’ the women. I also felt it wasn’t my ‘domain’ to discuss dental health issues.” (M17)*

*“[Oral health is an] extremely important part of primary health care. Midwives are in a pivotal position to educate and empower women to improve their health status.” (M19)*

*“In our scope of antenatal care, the information is important and comprehensive. It is surprising, how many women believe it is not safe to have dental treatment during pregnancy, and I have much greater confidence now when I discuss this important, and previously neglected, subject of oral care with the women.” (M22)*

Furthermore, participants discussed the significant need to raise awareness more broadly among all midwives and other health professional of their role in promoting and improving the oral health of pregnant women and their infants.

*“I think this should be made available for all midwives to participate in, very beneficial.” (M Feedback 1)*

*“I loved the program and really hope that it is something that we can offer to all midwives. I am so glad I had the opportunity to complete the package and I have already organised an oral health in pregnancy in-service for the midwives here at [our hospital].” (M Email 1)*

*“I am sure other maternal and child health nurses would find this program beneficial. Often, maternal and child health nurses have contact with women pregnant with their subsequent babies and discuss oral health for babies/toddlers/pre-schoolers at every visit from 4 months. It may be worth considering offering this program to these nurses as well as midwives given they could assist in referring women for dental services, and providing information.” (M Email 8)*

##### Feasibility of incorporating oral health into antenatal care

The majority of midwives said that it would be feasible to include oral health in their first antenatal booking visit. According to the midwives this was important to ensure the women were aware of the risk associated with poor oral health as early as possible and encourage good oral hygiene practices.

*“We need to be including oral hygiene into our antenatal practice along with good diet, exercise, vitamin supplements during pregnancy, non-smoking etc. It is an ideal time for Midwives to introduce the topic of oral hygiene and its importance.” (M20)*

*“It is information women should be aware of as early as possible in pregnancy so issues can be treated promptly and women are made aware as early as possible in the pregnancy of risk to their oral health and that of their baby.” (M8)*

*“[It is important to include oral health in] the first visit [as it] would include all clients…Other groups such as shared care clients would miss out as they do not visit the hospital after the first visit until 28 weeks.” (M8)*

Despite their busy schedules, many midwives explicitly mentioned that it would be easy to incorporate simple oral health questions and mouth checks into their first antenatal booking visit.

*“It doesn’t take long to ask the questions, and look in the mouth.” (M18)*

*“It can easily become another health promoting activity and potential problem identification task.” (M7)*

*“Even though we are all very busy in our antenatal clinics, it is feasible to include two simple questions as part of our first antenatal booking visit. This course has highlighted the importance of good oral hygiene during pregnancy to enable good outcomes for pregnant women and their unborn babies. It is an ideal time to educate the women about the importance of “Brushing their teeth” twice a day and to floss at least once a day. These things we do automatically are not always done by the women we see.” (M20)*

However, a few midwives did highlight that time constraints and competing health issues were still the main barriers to including oral health in the first antenatal booking visit.

*“There is so much information to go through …and already clients can become overwhelmed. I work with clients with addictions and mental health issues… adding length and added information… would be very stressful. I have decided it can wait till the second antenatal visit.” (M11).*

##### Intentions to change practice

Midwives acknowledged that they had the ideal opportunity to include oral health as part of their practice and a few midwives had already begun to incorporate oral health into their practice.

*“Thank you so much for putting this terrific learning program together. It has been a very positive learning journey for me. I am looking forward to implementing this information into the educational sessions our department facilitates for parents, both new and experienced as well as school students who come to us for education sessions.” (M8)*

*“Already I have organised an in-service for antenatal clinic midwives to address the deficit in our knowledge base of oral health in pregnancy.” (M 21)*

*“I now talk to women about oral care at their 1st visit and ask the questions set out in the study course.” (M22)*

### Confidence

There was a marked increase in the level of confidence of midwives to perform each of the oral health promotion tasks mentioned in the questionnaires (range 36.4-72.8%) after completing the MIOH education program. The greatest improvement was seen in relation to “*introducing the topic of oral health in antenatal care*”, *“incorporating oral health into the first antenatal booking visit”* and “*referring pregnant women to dental services*” (see Table 2). Table 3 shows a significant increase (51.5%, p < .0.001) in the mean confidence levels of midwives to perform the tasks post-training compared to pre-training.

**Table 2:** **Proportion of midwives who rated themselves as confident to perform tasks pre and post training and improvement observed**

**Oral Health Promotion**	**Confidence**		
**Item**	**Pre-training**	**Post-training**	**Change**
	**% (n)**	**% (n)**	%*
Conduct a visual mouth check on a pregnant woman	9.1(3)	57.6 (19)	48.5
Introduce the topic of oral health in antenatal care	24.2 (8)	97 (32)	72.8
Explain public dental policy on access to services by eligible pregnant women	24.2 (8)	63.9 (21)	39.9
Give advice about eligibility for public dental services to pregnant women	18.2 (6)	63.6 (21)	45.4
Find the nearest public dental service	42.4 (14)	78.8 (26)	36.4
Refer pregnant women to dental services	36.4 (12)	90.6 (29)	54.2
Incorporate oral health into the first antenatal booking visit	21.2 (7)	84.8 (28)	63.6

*Based on those midwives who responded ‘confident’ (rather than ‘somewhat’ or ‘not confident’).

**Table 3:** **Comparison of mean confidence levels of midwives pre and post training**

	**Pre-training**	**Post-training**	**Change**	**p-value**
	**%**	**%**	**%**	
Mean confidence level (SD)	25.1(11.16)	76.6(15.18)	51.5 ↑	*p* < 0.001

## Discussion

The role of antenatal care providers in promoting the oral health of pregnant women has been highlighted world-wide and more recently in Australia with the inclusion of oral health in the national antenatal care guidelines [29,30]. Midwives are appropriately placed to provide oral health assessment, information and referral during pregnancy and feel they have a role to play in oral health promotion [1,23,39,47-50]. Prior research in Australia has identified the educational gaps, needs and views of midwives in NSW which led to the development of the MIOH education program in an approach to fill the perceived gap [1,39,42].

In order to support the inclusion of oral health into midwifery practice in Victoria the current study tested and evaluated the usefulness of the Midwifery Initiated Oral Health (MIOH) online education program for informing antenatal practice among Victorian midwives. We explored Victorian midwives perspectives of the feasibility and their confidence to incorporate oral health promotion into the first antenatal booking visit.

### MIOH addresses an oral health knowledge gap

One of the significant themes raised by Victorian midwives was their limited prior education, knowledge and understanding of oral health promotion and the implications of poor oral health for pregnant women. This confirmed similar findings of preliminary research conducted in NSW [39]. The knowledge deficits of nurses and midwives do not appear to be unique to Australian education programs and have been reported internationally for example in Norway, North Carolina and Iran [47,51,52]. Victorian study participants were very receptive to the MIOH program and discussed the immense gains in knowledge of the implications of poor oral health for pregnant women that they received from participating in the education program. In particular, participation in the program raised awareness among Victorian midwives of the significance and importance oral health risks exacerbated during pregnancy; increased their knowledge on the impact of oral health on pregnancy outcomes and the influence of maternal oral health on infant oral health outcomes. Addressing this knowledge gap among midwives is the vital first step to facilitate the implementation of the current Australian antenatal care guidelines [30].

### Oral health is relevant to midwifery practice and antenatal care

It is clear from rich expressive responses that participating in the education program empowered midwives and provided them with new knowledge, confidence and practical skills and the ability to now recognise the significance of promoting oral health during pregnancy. Midwives discussed the relevance of oral health promotion to their practice, suggesting that other midwives and health professionals should complete the program. This concept of oral health as integral to general health is not new [53] and has been a major focus in other parts of the world. For example, in the United States there have been calls to action for the incorporation of oral health into perinatal health, not just limited to antenatal care, but considered through pregnancy, delivery and early childhood by all health professionals providing care from pregnancy to early childhood [28,36]. Furthermore, this supports a broader notion which proposes that to see real sustainable impacts on oral health requires a shift to integrating oral health and general health [54]. The entire primary health care system (dental and non-dental professionals) needs to be engaged, and consistent in their approach to oral health promotion [55-57]. This is especially important as there appears to be a lack of consensus among antenatal care providers and dentists regarding oral health care during pregnancy [58]. Incorporating oral health in antenatal care through midwives is just a beginning. Perhaps supporting a more broad reaching systems thinking approach is required, which looks at the health system in its entirety and the range of influential, interrelated sectors involved [59]. For example, involving partnerships between public and private sectors, tertiary providers and professional associations to ensure the inclusion of oral health in tertiary curriculums, national consensus statements outlining the key messages and guidelines for oral health risk assessments and oral health assessment by non-dental professionals (e.g. pharmacists, nurse, doctors, midwives, dietitians), as well as formalised referral pathways accessible between health professionals and public and private dental providers.

### Incorporating oral health into the first antenatal booking visit

Incorporating oral health into the first antenatal booking visit was consistently viewed as feasible by participating midwives and there was a significant improvement in their confidence to implement this. Midwives recognise their pivotal role in ensuring the wellbeing of mother and baby and the significance of incorporating oral health within their scope of practice. Many participants agreed that the booking visit was a prime opportunity and an ideal time to include simple oral health education and timely referral for the prevention of adverse oral health and wellbeing outcomes for the pregnant women and unborn child. This is despite the competing priorities and limited time available during consultations, and the importance of these as major barriers in both the current study and previously [39].

The language used by the midwives revealed an enthusiasm to apply what they had learnt in their practice and strong support for the inclusion of oral health promotion in the first antenatal booking visit. The enthusiasm and support expressed by midwives increases the potential for longer term, sustainable strategies which will broaden the scope of practice of midwives in Australia to incorporate oral health. Our findings indicate that participating Victorian midwives feel they have the capacity and responsibility to incorporate oral health promotion into antenatal care.

### The next steps for oral health promotion in antenatal care

The limited oral health educational opportunities available for Victorian midwives reflects an educational gap identified in NSW and other counties [47,60]. The MIOH education program provides midwives with the ‘how to include oral health promotion in the first antenatal booking visit’ and supports the implementation of the recommendation in the new national Australian antenatal care guidelines. Prospects and opportunities need to be sought to incorporate greater emphasis on oral health into midwifery curricula as a key component of antenatal care.

In Victoria the provision of the online oral health education program is supported by the policy of priority access to public dental services for pregnant women who have health care cards. However, further to these steps, strengthening of referral pathways between antenatal care providers and the local public and private dental services needs to be explored to support midwives to refer their clients for dental check-up and treatment during pregnancy. Work has already begun in one Victorian location to test an e-referral system which facilitates direct referral between midwives and the local public dental service.

As a result of this current study, further training has already been rolled out in Victoria including monitoring and detailed evaluation. This includes further enquiry and follow-up to explore whether the knowledge, confidence and skills gained though participation in the MIOH program are reflected and implemented in practice as well as discussion of referral pathway and relationships between various antenatal care providers and dental services. To further support and facilitate this initiative the MIOH program has been included in the Victorian Government Action Plan for Oral Health Promotion (2013–2017) [61]. Work is also being done to incorporate oral health assessment and referral into a clinical management information system used by many Victorian maternity services, as well as the early development of partnerships with Victorian universities for the inclusion of oral health in midwifery education. Currently, a trial is also underway in NSW examining the impact of the MIOH program on oral and pregnancy outcomes [62]. Further studies to assess the usefulness of such a program for other professional groups working in antenatal care would also be worthwhile.

### Strengths and limitations

A limitation of this study is that it was a pilot study with small participant number. It would be beneficial to follow-up midwives who took part in the education program to explore whether the participation has transferred into their clinical practice as well as the impacts for pregnant women. Purposive sampling is a non-representative sample as the sample is constructed for a specific purpose (such as including hospitals with high numbers of births). Participants self-selected to participate and therefore may represent a more receptive and motivated cohort rather that the general population of Victorian midwives. A strength of this study is its qualitative nature, with the inclusion of short-answer questionnaire responses paired with supporting emails and feedback forms. Although researchers were limited in their ability to probe and elaborate on perspectives (as in other qualitative methods such as interview or focus groups), the data still enabled the emergence of key themes and provided important insights into the thoughts and experiences of participating midwives.

## Conclusions

The unique role of midwives in oral health promotion is gaining real momentum in Australia and internationally. This study has demonstrated the usefulness and suitability of the MIOH education program to support Victorian midwives skills and confidence to undertake this role. Furthermore the study has highlighted study participants passion and enthusiasm toward oral health promotion and the feasibility of incorporating oral health promotion into antenatal practice, despite time constraints.

The established engagement with midwives, hospitals and health services across Victoria and NSW, the addition of oral health promotion to the national antenatal care guidelines and the developing role of midwives in oral health promotion provides the ideal opportunity to leverage off these inroads to continue to explore and define the role of antenatal health care professionals at a state and national level.

The foundations of a shift in oral health promotion within the public health system are beginning to be laid. To create change at a system level requires embedding oral health into national education systems, strengthening the evidence-base, influencing policy, raising awareness, building partnerships and links for effective and easy referral and expanding the scope of practice of antenatal care providers within the public health care systems. A commitment to a multilevel, multistrategic approach must be maintained.

## Abbreviations

DHSV: Dental Health Services Victoria
HFHS: Healthy Families, Healthy Smiles
MIOH: Midwifery Initiated Oral Health
NSW: New South Wales
VIC: Victoria
